# Phylogenetic Analysis of *Filifactor alocis* Strains Isolated from Several Oral Infections Identified a Novel RTX Toxin, FtxA

**DOI:** 10.3390/toxins12110687

**Published:** 2020-10-30

**Authors:** Jan Oscarsson, Rolf Claesson, Kai Bao, Malin Brundin, Georgios N. Belibasakis

**Affiliations:** 1 Division of Oral Microbiology, Department of Odontology, Umeå University, 90187 Umeå, Sweden; rolf.claesson@umu.se; 2 Division of Oral Diseases, Department of Dental Medicine, Karolinska Institutet, 14104 Huddinge, Sweden; kai.bao@ki.se (K.B.); george.belibasakis@ki.se (G.N.B.); 3 Division of Endodontics, Department of Odontology, Umeå University, 90187 Umeå, Sweden; malin.brundin@umu.se

**Keywords:** *Filifactor alocis*, oral infections, FtxA, multilocus sequence typing (MLST), phylogenetic tree

## Abstract

*Filifactor alocis* is a Gram-positive asaccharolytic, obligate anaerobic rod of the phylum Firmicutes, and is considered an emerging pathogen in various oral infections, including periodontitis. We here aimed to perform phylogenetic analysis of a genome-sequenced *F. alocis* type strain (ATCC 35896; CCUG 47790), as well as nine clinical oral strains that we have independently isolated and sequenced, for identification and deeper characterization of novel genomic elements of virulence in this species. We identified that 60% of the strains carried a gene encoding a hitherto unrecognized member of the large repeats-in-toxins (RTX) family, which we have designated as FtxA. The clinical infection origin of the *ftxA*-positive isolates largely varied. However, according to MLST, a clear monophylogeny was reveled for all *ftxA*-positive strains, along with a high co-occurrence of lactate dehydrogenase (*ldh*)-positivity. Cloning and expression of *ftxA* in *E. coli*, and purification of soluble FtxA yielded a protein of the predicted molecular size of approximately 250 kDa. Additional functional and proteomics analyses using both the recombinant protein and the *ftxA*-positive, and -negative isolates may reveal a possible role and mechanism(s) of FtxA in the virulence properties of *F.* *alocis*, and whether the gene might be a candidate diagnostic marker for more virulent strains.

## 1. Introduction

*Filifactor alocis* is a Gram-positive asaccharolytic, obligate anaerobic rod, belonging to the phylum Firmicutes. The species has recently been discovered in the oral microbiome via high-throughput sequencing methods and has successfully been cultivated in the laboratory. It is considered an emerging oral pathogen, with significant roles in the etiology of periodontal [1,2], peri-implantitis [3] and endodontic [4] infections. Currently, only the genome of one strain, ATCC 35896, has been sequenced (GenBank: CP002390), revealing a number of virulence properties consistent with the characteristics of a pathogen, such as neutrophil-activation protein A (NAPA), several proteases, and a calcium-binding acid repeat protein (CBARP) [5,6]. Interestingly, *F. alocis* was shown to manipulate neutrophils [7], and, in particular, their capability of forming neutrophil extracellular traps (NETs), phagosome maturation and reactive oxygen species (ROS) production were prevented, thus enhancing bacterial survival upon phagocytosis [8,9,10]. The repeats-in-toxins (RTX) family of toxins is a large family of exotoxins, in which most have been identified in Gram-negative bacteria, and they are typically encoded in gene operons also mediating their mode of extracellular release (type-I secretion) [11]. However, recent findings also support their presence in a number of Gram-positive organisms [11,12]. Examples of archetypal members of the RTX family are the alpha-hemolysin (HlyA) of extraintestinal pathogenic (ExPEC) *Escherichia coli* [13], the leukotoxin (LtxA) of the periodontal pathogen *Aggregatibacter actinomycetemcomitans* [14], and the adenylate cyclase bifunctonal toxin, CyaA, of the respiratory pathogen *Bordetella pertussis* [15]. We have in the present work screened the whole genome of the *F. alocis*-type strain ATCC 35896, for novel identification and deeper characterization of virulence elements. We have discovered that this strain encodes a hitherto unrecognized RTX toxin member, which we have here designated as “FtxA” for consistency with the nomenclature of other RTX toxin-gene encoding operons. In the present study, we have used the reference strain ATCC 35896, and our clinical collection of nine additional *F. alocis* strains, isolated from different oral infections, with the aim to further characterize the FtxA protein, and whether the gene encoding it may be conserved in phylogenetic lineage(s) of *F. alocis*, and hence might represent a candidate diagnostic marker for more virulent strains.

## 2. Results

### 2.1. Identification of A Putative RTX Toxin, FtxA, Encoded by F. alocis Reference Strain ATCC 35896

**Figure 1:**
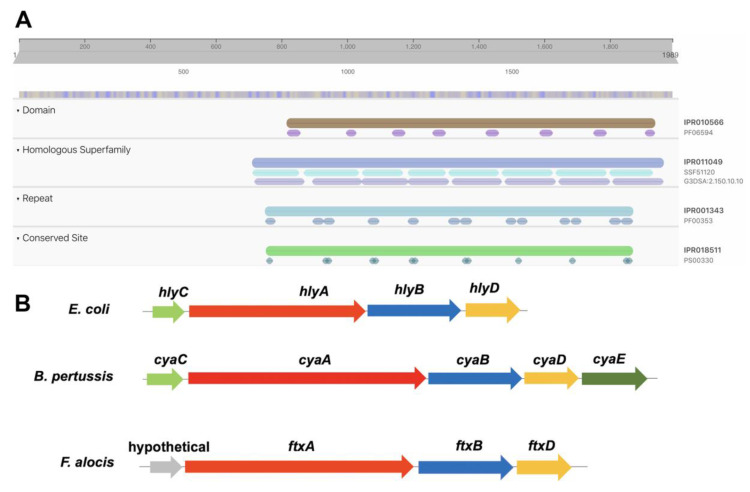
(**A**) Overlapping conserved domains in the FtxA protein sequence, deduced using InterPro. The C-terminal portion displays homology to the Hemolysin-type calcium binding-related domain (IPR010566), and the serralysin-like metalloprotease superfamily (IPR011049), and contains repeats-in-toxins (RTX) calcium-binding nonapeptide repeats (IPR001343), and Hemolysin-type calcium-binding conserved sites (IPR018511). (**B**) One archetypal RTX gene cluster (*hlyCABD*) encodes the *E. coli* alpha-hemolysin, HlyA. HlyA is synthesized as an inactive protoxin, ProHlyA, which is post-translationally activated in two steps, first via HlyC-directed acylation in the cytoplasm, and then by binding Ca^2+^ in the extracellular medium [13]. The *cyaE* gene of *B. pertussis* encodes a homologue to *E. coli tolC* and plays a critical role in the secretion of CyaA [11]. We identified no equivalent to an HlyC-homologue in the *F. alocis* genomes. The hypothetical protein encoded upstream of *ftxA* exhibits no apparent homology to *hlyC* or to other known proteins.

*In silico* analysis of the genome of the reference strain ATCC35896/CCUG 47790 revealed that it encodes a putative RTX toxin family member (ADW16141.1; HMPREF0389_01695; “Type I secretion target GGXGXDXXX repeat”), with a theoretical molecular weight of 223 kDa, based on the length of the protein (1989 amino acids). According to BLAST, its closest homologue in other species appears to be a Ca^2+^-binding, RTX-related protein of the Gram-positive bacterium *Eubacterium yurii* strain ATCC 43714 (SKC68031), which was ≈35% identical at the amino acid level over the entire protein sequence. No conserved functional domain was detected in the N-terminal part of FtxA (amino acids 1–749). However, it was found to contain a GXSXXG motif at amino acids 412–417, which has been reported to be associated with lipase, esterase, phosphatase, and protease activity [16,17]. According to secondary structure prediction using Phyre^2^, amino acids 301–491 of FtxA display 39% amino acid identity to the RTX-related, and iron-regulated protein FrpC (6SJX_A) in *Neisseria meningitidis*, and it was observed that this region of FtxA is similar to a self-processing module found in other RTX proteins, such as FrpC [18]. The C-terminal portion (amino acids 750–1989) was found to contain several (*n* = 16) Ca^2+^-binding repeat domains, and proteolytic domains (i.e., serralysin-like metallo-protease), respectively (Figure 1A). Phyre^2^ predicted domains near the C-terminus (amino acids 1655–1948) to belong to the β-roll superfamily, exhibiting single-stranded right-handed beta-helix folds. No C-terminal export signal was detected in the FtxA protein sequence using either SignalP or SecretomeP.

Concomitantly, especially the C-terminal part of FtxA displayed homology to several known RTX toxins, such as the *B. pertussis* adenylate cyclase toxin (CyaA; 25% amino acid identity), and to the *A. actinomycetemcomitans* leukotoxin (LtxA; 30%). The RTX protein of *F. alocis* has in the present work been designated as *Filifactor* Toxin A (FtxA), and it appears to be encoded in a three-gene operon, with a type-I secretion ATP:ase, *ftxB* (EFE27661; HMPREF0389_01580), and a type-I secretion membrane fusion protein, *ftxD* (EFE27662; HMPREF0389_01581). This is typical of RTX toxin-encoding operons, with the exception that it lacks an activating lysine-acyltransferase (Figure 1B). *E. yurii* ATCC 43714 was also found to exhibit this operon structure, also encoding equivalents to HlyB (SKC68044) and HlyD (SKC68061).

### 2.2. Isolation of F. alocis Clinical Strains and PCR Screening for ftxA

To assess whether *ftxA* is a conserved gene in *F. alocis*, this species was isolated at the clinical oral microbiology laboratory, Dental School, from clinical samples collected from nine patients as described in Materials and Methods (Table 1).

Strain 624B-08U had been isolated earlier, in a clinical sample from periodontal pockets, subject to bacterial diagnostics, and which contained >90% *F. alocis* out of the total viable count (TVC). According to PCR, *ftxA* was encoded by five of the ten tested strains (data not shown). To corroborate the PCR results, and for subsequent multilocus sequence typing (MLST), all nine strains that had been isolated at the clinical laboratory were subject to whole-genome sequencing. Extraction of the genome sequence data essentially confirmed the PCR-findings, with highly conserved FtxA protein sequences, encoded in apparent *ftxABD* operons. However, one of the *ftxA*-negative strains according to PCR, 854G-16U, was in fact found to encode an FtxA homologue. Relative to the ATCC 35896 FtxA protein, it was only ≈46% identical at the amino acid sequence level, consistent with sequence variability among the FtxA proteins in *F. alocis*. Taken together, *ftxA* was carried by six of the ten tested strains and is therefore not a universal property of this bacterium.

### 2.3. Isolation of F. alocis Clinical Strains and PCR Screening for ftxA

**Figure 2:**
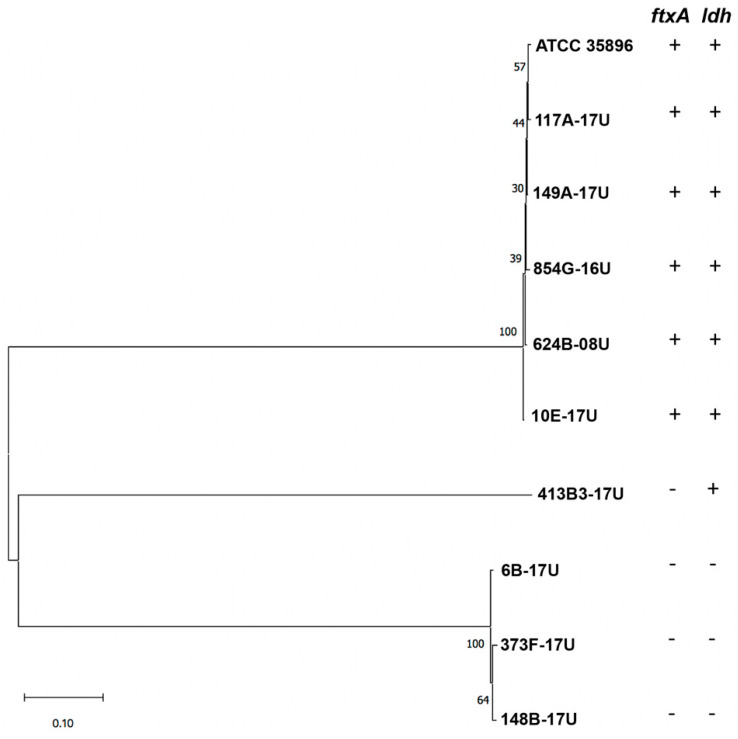
Phylogenetic relationships among the ten *F. alocis* strains, based on multilocus sequence typing (MLST) analysis as described in Materials and Methods. The tree with the highest log likelihood is shown. The percentages of trees in which the associated strains clustered together is shown next to the branches. The tree is drawn to scale, with branch lengths measured in the number of substitutions per site. The *ftxA* and *ldh* genotypes, respectively, are indicated.

To analyze the evolutionary distances between the ten *F. alocis* strains, including the reference strain ATCC 35896, MLST was next performed, using a novel scheme with eight genes as described in Materials and Methods. It was observed that the *ldh* gene was absent in three of the four strains lacking *ftxA* (Table 1). Cluster analysis, using the eight concatenated sequences, showed that the *ftxA*-positive, and *ftxA*-negative strains represented distinct monophyletic groups (Figure 2). Moreover, the *ldh*-negative strains represented a separate branch.

### 2.4. Expression and Purification of Purified Recombinant FtxA

**Figure 3:**
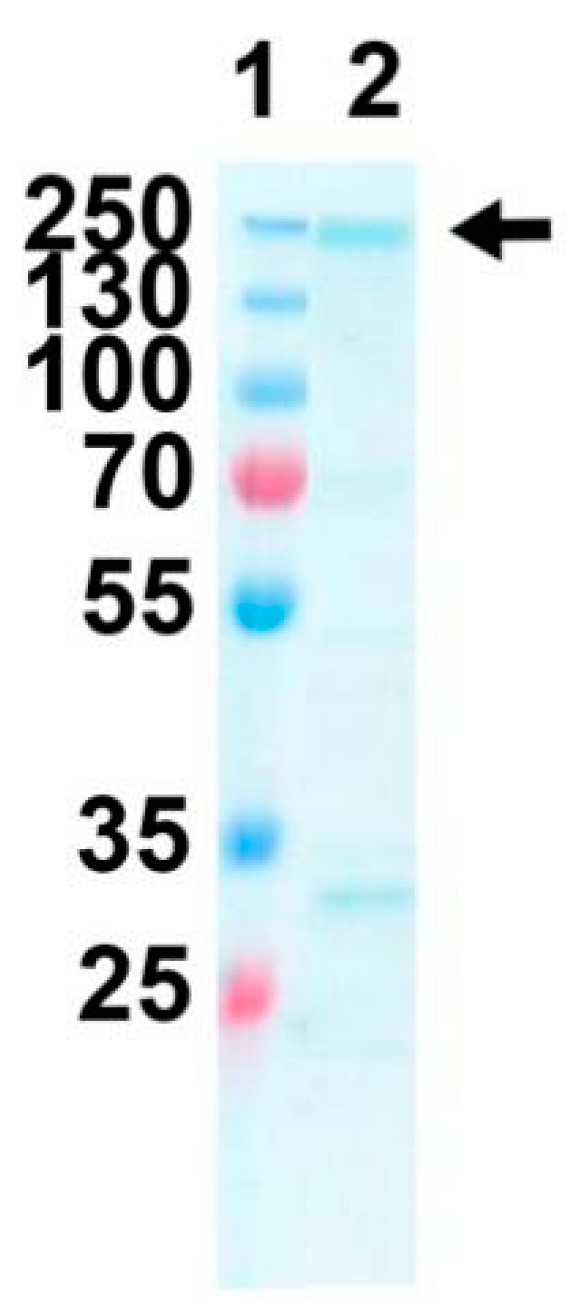
Coomassie-staining reveals purified recombinant FtxA as a high molecular weight band near 250 kDa (lane 2). A molecular weight marker was loaded in lane 1, and the molecular sizes (kDa) of the individual protein bands are indicated.

As a starting point for future functional assessments of FtxA, the *ftxA* gene was cloned and expressed in *E. coli*, and the protein was subsequently purified as described in Materials and Methods. Using these procedures, soluble FtxA was obtained at a concentration of approximately 0.2 mg/mL (Figure 3). Hence, it was concluded that FtxA could be obtained as a recombinant protein.

## 3. Discussion

In this work, we have discovered a novel exotoxin, here designated FtxA, belonging to the RTX family, and encoded by the emerging oral pathogen *F. alocis*, which is associated with multiple oral infections including periodontitis. The mere presence of FtxA and its identification as a new member of the RTX family of toxins is of particular importance in the oral domain. That is because the only other oral species hitherto known to possess exotoxins is *A. acinomycetemcomitans*, which can express both an RTX leukotoxin (LtxA), and a cytolethal distending toxin (CDT) [14,19]. The present discovery makes *F. alocis* the second oral species known to encode a protein exotoxin, and FtxA the third such toxin to be identified in a resident oral cavity-derived species. The apparent three-gene operon pattern, *ftxABD*, is similar to the *rtx* gene cluster of *Streptococcus sanguinis*, which also contains only the HlyA, B, and D equivalent [12], and would be consistent with a different mode of activation and extracellular release of RTX toxins in Gram-positive bacteria, lacking the outer membrane, and thus by definition type-I secretion. RTX toxins released by type-I secretion systems typically contain a C-terminal export signal of approximately 60 amino acids, although no consensus sequence has been observed within these signals [20]. Whether FtxA contains such an export signal is not yet known. The relevance of RTX domains in FtxA, with 25% amino acid identity to CyaA, for FtxA secretion also remains to be assessed. According to the secondary structural prediction, a β-roll motif can theoretically be formed. Hence, it could be speculated that FtxA secretion might be driven by the calcium gradient between the bacterial cytoplasm (below μM CaCl_2_) and the saliva (around mM CaCl_2_), i.e., in a manner similar to the disorder-to-order transition observed for CyaA [21].

The present observation that only 60% of the tested *F. alocis* strains encoded FtxA indicates that RTX exotoxin production is not a universal property of this organism. Hence, different pathotypes with disparate pathogenic strategies might exist within this species, similar to in, e.g., *E. coli*, where RTX exotoxin production is restricted to the extra-intestinal (ExPEC), and enterohemorrhagic (EHEC) pathotypes [11,15]. Albeit the clinical origin of the *ftxA*-positive isolates largely varied, and the FtxA protein sequence in one case (strain 854G-16U) was rather different, the grouping of them in one monophyletic branch in our novel MLST analysis scheme, and the high co-occurrence of the *ldh* gene, supports the notion that this is a group of genetically similar strains. Although a limited number of *F. alocis* strains were assessed in the present study, the recognition of a genotype encoding *ftxA* may appear useful in future diagnostic approaches, to detect the carriage of strains of this species with potentially enhanced virulence. Alignment of the entire genomes of the ten *F. alocis* strains will be helpful in delineating a pan-genome, and the core and accessory genomes of this species, for deeper characterization of its arsenal of virulence elements.

Although there was limited conservation of FtxA in relation to other RTX toxins, a relatively closely related Gram-positive organism, *E. yurii* [22], was found to encode the most similar known protein in the National Center for Biotechnology Information (NCBI) database (≈35% amino acid identity). Similar to *F. alocis*, this species has been reported to be associated with various oral, including periodontal [23], and endodontic [24] infections, and it has also been identified in peri-implantitis lesions [25]. With their overlapping niches, it cannot be excluded that FtxA and the *E. yurii* RTX toxin might play a similar role(s) in the virulence strategies of these species. This, however, remains to be investigated.

For future activity studies, we have further cloned and purified FtxA, to confirm that it can be expressed as a recombinant protein and to confirm its predicted molecular size. The observation that FtxA contains a region in the N-terminal portion, which is similar to a module found in several other RTX proteins, important for Ca^2+^-dependent autocatalytic cleavage [18], suggests the possibility that FtxA may be subjected to self-processing in a similar manner. The motif GXSXXG, recognized in the N-terminal part of FtxA is known as a nucleophilic elbow in the α/β hydrolase fold protein superfamily, and is associated with hydrolytic enzymes of widely different functions, including esterases [26,27]. This would suggest the possibility that FtxA might exhibit hydrolytic activity. However, in FtxA, the Serin residue (Ser414) of this motif is located in a structural segment that does not resemble a nucleophilic elbow, and no other residues (His, Asp), which could be part of the catalytic triad [26], are located in its neighborhood, based on the homology model. In addition, the segment comprising residues 301 to 491 appear to be very different from the usual α/β hydrolase fold.

The expression and purification procedures are presently being optimized to obtain high-quality purified recombinant FtxA to allow for more specific functional studies. Interestingly, FtxA, according to its GenBank database definition, was recently identified among a number of additional gene products in extracellular vesicles (EVs) released by *F. alocis* ATCC 35896 [28]. Whether it might have played a role in the observed immunostimulatory effects of the EVs on human monocytic and oral keratinocyte cell lines is not known. However, the well-documented pathogenic actions of *F. alocis* on the host [1,29], particularly on the subversion of neutrophil migration and function [10,30], draw the attention to a potential role of FtxA for future studies.

At this early stage, the particular roles of this potential toxin in pathogenicity of *F. alocis* in periodontal disease cannot be inferred. To continue to understand the role of FtxA in the virulence characteristics of *F. alocis* and/or in the resident biofilm ecology, additional functional and proteomics analyses using both the recombinant protein and the *ftxA*-positive, and -negative isolates, and genetic analysis of the yet uncharacterized N-terminal will shed more light to its potential virulence properties and etiopathological roles in oral, including periodontal disease.

## 4. Conclusions

The identification of a novel RTX toxin, FtxA, encoded by the emerging oral pathogen *Filifactor alocis,* has extended the concept of exotoxin production in the oral domain, and may provide a foundation for future efforts to disclose pathogenicity mechanisms, to diagnose and prevent oral infections such as periodontal disease.

## 5. Materials and Methods

### 5.1. Isolation of F. alocis Strains, and Culturing Conditions

The reference *F. alocis* strain was purchased from the Cultural Collection of the University of Gothenburg (CCUG), i.e., CCUG 47790. This strain is also known as ATCC 35896 and was originally isolated from gingival sulci of patients with gingivitis or periodontitis [31,32]. We further characterized another nine *F. alocis* strains in this study. Among those, six were isolated from samples collected from periodontal pockets, while the remaining originated in infected dental root canals (Table 1). The carriers of the collected *F. alocis* were between 15 and 67 years old. The samples were collected with paper points and transported to the clinical laboratory, Dental School, Umeå, Sweden, in anaerobic transport mediums: VMGAIII [33] (periodontal pocket samples), or Fluid Thioglycollate Medium (FTM; Oxoid Ltd., Basingstoke, Hampshire, UK) (root canal samples). After disintegration of the paper points by vortex agitation, the samples were serially diluted and spread on blood agar (BGA; 5% defibrinated horse blood, 5 mg hemin/L, 10 mg Vitamin K/L, Columbia agar base; Oxoid Ltd., Basingstoke, Hampshire, UK), or fastidious anaerobe agar (FAA; Neogen^®^, Heywood, UK). For *F. alocis* cultivation, the agar plates were incubated in an anaerobic environment (10% H_2_, 5% CO_2_, 85% N_2_) at 37 °C for seven days and subsequently examined for presence of *F. alocis*. Gliding and translucent colonies, displaying rod-shaped cell morphology were considered as presumptive *F. alocis* and were isolated. In a following step, arginine-positive isolates, identified by using Nessler’s reagent (Sigma-Aldrich, St. Louis, MO, USA), were confirmed as *F. alocis* by procedures described earlier [34], i.e., Maldi-TOF, and/or 16s rRNA-gene sequencing. For experimental purposes, the strains were cultivated on BGA or FAA plates, incubated in anaerobic conditions at 37 °C as described above.

### 5.2. DNA Isolation and Polymerase Chain Reaction-Based Characterization

DNA templates for PCR analysis were obtained by boiling a loopful of fresh bacterial colonies in water. F. alocis genomic DNA to be used in whole-genome sequencing was isolated using the QIAamp^®^ DNA Mini kit (Sigma-Aldrich, St. Louis, MO, USA), following the manufacturer’s instructions with the exception that DNA was eluted using 10 mM Tris-HCl, pH 8.5. PCR reaction mixtures were prepared using illustra™ PuReTaq™ Ready-To-Go™PCR beads (GF Healthcare, Buckinghamshire, UK). The species identity of *F. alocis* isolates was confirmed by sequencing >95% of the 16S rRNA gene after amplification with primers 9F (5′GAGTTTGATYMTGGCTCAG-3′) and 1541R (5′-AAGGAGGTGWTCCARCC-3′) as described [35]. The amplified gene fragments were sequenced by Eurofins Genomics (Ebersberg, Germany). The nucleotide sequences were searched with BLAST for similarities with known sequences in GenBank and are available at the NCBI database (www.ncbi.nlm.nih.gov). To confirm the results, the *F. alocis* 16s rRNA gene was qPCR-amplified with forward primer (5′-CAGGTGGTTTAACAAGTTAGTGG-3′) and reverse primer (5′-CTAAGTTGTCCTTAGCTGTCTCG-3′), producing a PCR amplicon of 594 base pairs (bp), as described earlier [36]. The *ftxA* gene was amplified by PCR as a 798-bp internal fragment using the forward primer (5′-GGCTCAGATACCTACTTCTTC-3′), and reverse primer (5′-GAAGGCTATGATTTGATTGTTTCC-3′). PCR cycling conditions were 95 °C for 1 min, followed by 35 cycles of 95 °C for 30 s, 54 °C for 30 s, 72 °C for 1 min and then finally 72 °C for 7 min.

### 5.3. Whole-Genome Sequencing

Genomic DNA samples from strains 854G-16U, 117A-17U, 149A-17U, 624B-08U, 373F-17U, 6B-17U, 10E-17U, 413B3-17U, and 148B-17U were prepared as described above, and sent to MicrobesNG (Birmingham, UK) for whole-genome sequencing. The obtained trimmed reads were annotated with Rapid Annotation using Subsystem Technology (RAST) (rast.nmpdr.org). The genome of the prototype strain ATCC 35896 (GenBank accession CP002390) was used as the reference.

### 5.4. In Silico Analysis and Protein Domain Prediction

Gene sequences of *ftxA*, and those encoding housekeeping proteins to be utilized in MLST, were extracted from the whole-genome sequencing data. The *ftxA* and housekeeping gene sequences identified in the present work will be deposited in the European Nucleotide Archive (http://www.ebi.ac.uk/ena). InterPro [37], available via the European Bioinformatics Institute (EMBL-EBI) at https://www.ebi.ac.uk/interpro/ was used to identify conserved domains in protein sequences. The Phyre^2^ web portal for protein modeling, prediction and analysis [38], available at http://www.sbg.bio.ic.ac.uk/~phyre2 was used for secondary structure prediction of FtxA. SignalP 5.0 and SecretomeP 2.0a (https://services.healthtech.dtu.dk/) was used for signal peptide prediction in the FtxA amino acid sequence.

### 5.5. Multilocus Sequence Typing (MLST) and Phylogenetic Analysis

Eight full-length genes encoding housekeeping proteins of the reference strain, ATCC 35896 were used in MLST: adenylate kinase (*adk*; WP_014262819), atp synthase (*atpG*; WP_014262442), lactate dehydrogenase (*ldh*; WP_014262939), recombinase (*recA*; WP_014261951.1), chaperonin (*groEL*; WP_014263140), molecular chaperone (*dnaK*; WP_014262200.1), DNA gyrase (*gyrA*; WP_014261695), and d-alanine—d-alanine ligase (*ddl*; WP_014261780). The gene sequences were selected based on the typing schemes of related Gram-positive bacteria such as *Clostridium spp*, which were available in the Public databases for molecular typing and microbial genome diversity (pubmlst.org). A cluster analysis of the ten *F. alocis* strains used in the present work was conducted, based on concatenated sequences in the order *adk, atpG, ldh, recA, groEL, dnaK, gyrA, and ddl*. Evolutionary analyses were conducted using Molecular Evolutionary Genetics Analysis (MEGA) X software (version 10), available at http://www.megasoftware.net [39,40]. Evolutionary history was inferred using the Maximum-Likelihood method and Tamura-Nei model, with 1000 bootstrap replicates [41]. There was a total of 10716 nucleotide positions used in the final dataset.

### 5.6. Cloning and Expression of Recombinant FtxA

Standard cloning procedures were used by the Protein Expertise Platform (PEP), at the Chemical Biological Center (KBC), Umeå University. The *ftxA* gene was amplified by PCR using Phusion^TM^ DNA polymerase (Thermo Fisher Scientific, Waltham, MA, USA) in two portions using forward A (5′-gtaacacctgcgaaccATGAAAGAACTAAGTCTGGAAATG-3′), and reverse A (5′-ATTCGGAATTCTCTTTGTCTTG-3′) primers, and forward B (5′- CAAGACAAAGAGAATTCCGAAT-3′), and reverse B (5′-gtaaaggtaccTTACATTTGTTTTGCCCAGAAG-3′) primers, respectively. The primer sequences were designed based on the strain ATCC 35896 genome. The non-capitalized bases indicate sequences added to allow cloning into the expression vector. The PCR products were ligated, and cloned into the expression vector pET-His1a [42]. For this, OverExpress™ C41(DE3) *E. coli* cells (Lucigen, Middleton, WI, USA) were used, and which were cultivated in Luria–Bertani (LB) broth or on LB broth solidified with 1.5% (*w/v*) agar. Kanamycin (final concentration 50 mg/L) was used to select for carriage of the vector. Expression of His-Tagged FtxA was induced by using 0.4 mM isopropyl β-d-1-thiogalactopyranoside (IPTG), and soluble protein was then purified on a Nickel-nitrilotriacetic acid (Ni-NTA) column (Thermo Fisher Scientific, Waltham, MA, USA). No refolding in the presence of Ca^2+^ was carried out. The His-Tag was removed using Tobacco Etch Virus (TEV) protease (kindly provided by the PEP).

### 5.7. Ethical Considerations

All procedures were conducted in accordance with the guidelines of the local ethics committee at the Medical Faculty of Umeå University, which are in accordance with the Declaration of Helsinki (64th WMA General Assembly, Fortaleza, October 2013).

### 5.8. Image Procession

Images for figures were assembled using Adobe Photoshop (version CS6; Adobe, San Jose, CA, USA), or PowerPoint (version 16; Microsoft, Redmond, WA, USA).

## Figures and Tables

**Table 1:** Sources of the clinical *F. alocis* strains, isolated and used in the present work, and their respective *ftxA* and *ldh* genotypes.

Strain	Source	*ftxA*-Positive (+) or Negative (−)	*ldh*-Positive (+) or Negative (−)
854G-16U	apical periodontitis, fistula	+	+
117A-17U	periodontitis	+	+
149A-17U	periodontitis	+	+
624B-08U	acute necrotizing ulcerative gingivitis (ANUG)	+	+
373F-17U	peri-implantitis	−	−
6B-17U	root canal	−	−
10E-17U	root canal	+	+
413B3-17U	periodontitis	−	+
148B-17U	periodontitis	−	−

## References

[1] Aruni W., Chioma O., Fletcher H.M. (2014). *Filifactor**alocis*: The Newly Discovered Kid on the Block with Special Talents. J. Dent. Res..

[2] Greenwood D., Afacan B., Emingil G., Bostanci N., Belibasakis G.N. (2020). Salivary Microbiome Shifts in Response to Periodontal Treatment Outcome. Proteom. Clin. Appl..

[3] Sanz-Martin I., Doolittle-Hall J., Teles R.P., Patel M., Belibasakis G.N., Hammerle C.H.F., Jung R.E., Teles F.R.F. (2017). Exploring the microbiome of healthy and diseased peri-implant sites using Illumina sequencing. J. Clin. Periodontol..

[4] Zehnder M., Rechenberg D.K., Thurnheer T., Luthi-Schaller H., Belibasakis G.N. (2017). FISHing for gutta-percha-adhered biofilms in purulent post-treatment apical periodontitis. Mol. Oral Microbiol..

[5] Aruni A.W., Roy F., Fletcher H.M. (2011). *Filifactor**alocis* has virulence attributes that can enhance its persistence under oxidative stress conditions and mediate invasion of epithelial cells by *Porphyromonas*
*gingivalis*. Infect. Immun..

[6] Aruni A.W., Roy F., Sandberg L., Fletcher H.M. (2012). Proteome variation among *Filifactor*
*alocis* strains. Proteomics.

[7] Uriarte S.M., Edmisson J.S., Jimenez-Flores E. (2016). Human neutrophils and oral microbiota: A constant tug-of-war between a harmonious and a discordant coexistence. Immunol. Rev..

[8] Armstrong C.L., Klaes C.K., Vashishta A., Lamont R.J., Uriarte S.M. (2018). *Filifactor**alocis* manipulates human neutrophils affecting their ability to release neutrophil extracellular traps induced by PMA. Innate Immun..

[9] Edmisson J.S., Tian S., Armstrong C.L., Vashishta A., Klaes C.K., Miralda I., Jimenez-Flores E., Le J., Wang Q., Lamont R.J. (2018). *Filifactor**alocis* modulates human neutrophil antimicrobial functional responses. Cell Microbiol..

[10] Miralda I., Vashishta A., Uriarte S.M. (2019). Neutrophil Interaction with Emerging Oral Pathogens: A Novel View of the Disease Paradigm. Adv. Exp. Med. Biol..

[11] Linhartova I., Bumba L., Masin J., Basler M., Osicka R., Kamanova J., Prochazkova K., Adkins I., Hejnova-Holubova J., Sadilkova L. (2010). RTX proteins: A highly diverse family secreted by a common mechanism. FEMS Microbiol. Rev..

[12] Xu P., Alves J.M., Kitten T., Brown A., Chen Z., Ozaki L.S., Manque P., Ge X., Serrano M.G., Puiu D. (2007). Genome of the opportunistic pathogen *Streptococcus sanguinis*. J. Bacteriol..

[13] Uhlin B.E., Oscarsson J., Wai S.N., Morabito S. (2014). Haemolysins. Pathogenic Escherichia Coli: Molecular and Cellular Microbiology.

[14] Johansson A. (2011). *Aggregatibacter**actinomycetemcomitans* leukotoxin: A powerful tool with capacity to cause imbalance in the host inflammatory response. Toxins.

[15] Ristow L.C., Welch R.A. (2019). RTX Toxins Ambush Immunity’s First Cellular Responders. Toxins.

[16] Derewenda Z.S., Sharp A.M. (1993). News from the interface: The molecular structures of triacylglyceride lipases. Trends Biochem. Sci..

[17] Jobin M.C., Martinez G., Motard J., Gottschalk M., Grenier D. (2005). Cloning, purification, and enzymatic properties of dipeptidyl peptidase IV from the swine pathogen *Streptococcus*
*suis*. J. Bacteriol..

[18] Osicka R., Prochazkova K., Sulc M., Linhartova I., Havlicek V., Sebo P. (2004). A novel “clip-and-link” activity of repeat in toxin (RTX) proteins from gram-negative pathogens. Covalent protein cross-linking by an Asp-Lys isopeptide bond upon calcium-dependent processing at an Asp-Pro bond. J. Biol. Chem..

[19] Belibasakis G.N., Maula T., Bao K., Lindholm M., Bostanci N., Oscarsson J., Ihalin R., Johansson A. (2019). Virulence and Pathogenicity Properties of *Aggregatibacter*
*actinomycetemcomitans*. Pathogens.

[20] Masi M., Wandersman C. (2010). Multiple signals direct the assembly and function of a type 1 secretion system. J. Bacteriol..

[21] O’Brien D.P., Perez A.C.S., Karst J., Cannella S.E., Enguene V.Y.N., Hessel A., Raoux-Barbot D., Voegele A., Subrini O., Davi M. (2018). Calcium-dependent disorder-to-order transitions are central to the secretion and folding of the CyaA toxin of *Bordetella*
*pertussis*, the causative agent of whooping cough. Toxicon.

[22] Sizova M.V., Chilaka A., Earl A.M., Doerfert S.N., Muller P.A., Torralba M., McCorrison J.M., Durkin A.S., Nelson K.E., Epstein S.S. (2015). High-quality draft genome sequences of five anaerobic oral bacteria and description of *Peptoanaerobacter*
*stomatis* gen. nov., sp. nov., a new member of the family Peptostreptococcaceae. Stand. Genom. Sci..

[23] Elabdeen H.R., Mustafa M., Hasturk H., Klepac-Ceraj V., Ali R.W., Paster B.J., Van Dyke T., Bolstad A.I. (2015). Subgingival microbial profiles of Sudanese patients with aggressive periodontitis. J. Periodontal. Res..

[24] Hsiao W.W., Li K.L., Liu Z., Jones C., Fraser-Liggett C.M., Fouad A.F. (2012). Microbial transformation from normal oral microbiota to acute endodontic infections. BMC Genom..

[25] Koyanagi T., Sakamoto M., Takeuchi Y., Ohkuma M., Izumi Y. (2010). Analysis of microbiota associated with peri-implantitis using 16S rRNA gene clone library. J. Oral. Microbiol..

[26] Ollis D.L., Cheah E., Cygler M., Dijkstra B., Frolow F., Franken S.M., Harel M., Remington S.J., Silman I., Schrag J. (1992). The α/β hydrolase fold. Protein Eng..

[27] Udatha D.B., Madsen K.M., Panagiotou G., Olsson L. (2015). Multiple nucleophilic elbows leading to multiple active sites in a single module esterase from *Sorangium*
*cellulosum*. J. Struct. Biol..

[28] Kim H.Y., Lim Y., An S.J., Choi B.K. (2020). Characterization and immunostimulatory activity of extracellular vesicles from *Filifactor*
*alocis*. Mol. Oral. Microbiol..

[29] Wang Q., Jotwani R., Le J., Krauss J.L., Potempa J., Coventry S.C., Uriarte S.M., Lamont R.J. (2014). *Filifactor**alocis* infection and inflammatory responses in the mouse subcutaneous chamber model. Infect. Immun..

[30] Vashishta A., Jimenez-Flores E., Klaes C.K., Tian S., Miralda I., Lamont R.J., Uriarte S.M. (2019). Putative Periodontal Pathogens, *Filifactor*
*alocis* and *Peptoanaerobacter*
*stomatis*, Induce Differential Cytokine and Chemokine Production by Human Neutrophils. Pathogens.

[31] Cato E.P., Moore L.V., Moore W.E.C. (1985). *Fusobacterium**alocis* sp. nov. and *Fusobacterium*
*sulci* sp. nov. from the human gingival sulcus. Int. J. Syst. Bacteriol..

[32] Jalava J., Eerola E. (1999). Phylogenetic analysis of *Fusobacterium*
*alocis* and *Fusobacterium*
*sulci* based on 16S rRNA gene sequences: Proposal of *Filifactor*
*alocis* (Cato, Moore and Moore) comb. nov. and *Eubacterium*
*sulci* (Cato, Moore and Moore) comb. nov. Int. J. Syst. Bacteriol..

[33] Möller A.J. (1966). Microbiological examination of root canals and periapical tissues of human teeth. Methodological studies. Odontol. Tidskr..

[34] Claesson R., Sjögren U., Esberg A., Brundin M., Granlund M. (2017). *Actinomyces**radicidentis* and *Actinomyces*
*haliotis*, coccoid *Actinomyces* species isolated from the human oral cavity. Anaerobe.

[35] Bahrani-Mougeot F.K., Paster B.J., Coleman S., Ashar J., Barbuto S., Lockhart P.B. (2008). Diverse and novel oral bacterial species in blood following dental procedures. J. Clin. Microbiol..

[36] Siqueira J.F., Rocas I.N. (2003). Detection of *Filifactor*
*alocis* in endodontic infections associated with different forms of periradicular diseases. Oral. Microbiol. Immunol..

[37] Mitchell A.L., Attwood T.K., Babbitt P.C., Blum M., Bork P., Bridge A., Brown S.D., Chang H.Y., El-Gebali S., Fraser M.I. (2019). InterPro in 2019: Improving coverage, classification and access to protein sequence annotations. Nucleic Acids Res..

[38] Kelley L.A., Mezulis S., Yates C.M., Wass M.N., Sternberg M.J. (2015). The Phyre2 web portal for protein modeling, prediction and analysis. Nat. Protoc..

[39] Kumar S., Stecher G., Li M., Knyaz C., Tamura K. (2018). MEGA X: Molecular Evolutionary Genetics Analysis across Computing Platforms. Mol. Biol. Evol..

[40] Stecher G., Tamura K., Kumar S. (2020). Molecular Evolutionary Genetics Analysis (MEGA) for macOS. Mol. Biol. Evol..

[41] Tamura K., Nei M. (1993). Estimation of the number of nucleotide substitutions in the control region of mitochondrial DNA in humans and chimpanzees. Mol. Biol. Evol..

[42] Bogomolovas J., Simon B., Sattler M., Stier G. (2009). Screening of fusion partners for high yield expression and purification of bioactive viscotoxins. Protein Expr. Purif..

